# Islamic law perspectives and social experiences on stigma toward disabled people in Indonesia

**DOI:** 10.3389/fsoc.2025.1479243

**Published:** 2025-03-12

**Authors:** Muktashim Billah, Abdul Qadir Gassing, Muammar Bakry, Kurniati Kurniati, Abdul Wahid Haddade, Darussalam Syamsuddin, La Ode Ismail Ahmad, Ahmad Harakan

**Affiliations:** ^1^Department of Family Law, Universitas Muhammadiyah Makassar, Makassar, Indonesia; ^2^Graduate Program, Universitas Islam Negeri Alauddin Makassar, Makassar, Indonesia; ^3^Doctoral School of International Relations and Political Science, Corvinus University of Budapest, Budapest, Hungary

**Keywords:** disabled people, Islamic law perspectives, social experiences, Maqasid al-Shariah, stigma

## Abstract

**Introduction:**

Stigmatization of disabled individuals remains a significant issue in Indonesia, particularly in Makassar, despite the existence of legal protections. This issue is further complicated by the intersection of religious and cultural norms, especially in Muslim-majority contexts. Islamic law (Maqāṣid al-Sharı̄'ah) provides a framework for dignity, intellect, and social justice, yet societal perceptions often contradict these principles. This study investigates how social stigma—manifested through harassment, insults, and negative labeling—aligns or conflicts with Islamic teachings on compassion and inclusion.

**Methods:**

A mixed-methods approach was employed, combining qualitative interviews with key informants and quantitative survey data from 400 respondents, sampled using the Taro Yamane formula. The study examines the prevalence of stigma against disabled people and explores the role of Islamic perspectives in shaping societal attitudes.

**Results:**

Findings reveal that stigma against disabled individuals in Makassar is deeply embedded in social structures, often reinforcing their marginalization. While Islamic teachings promote inclusivity and protection of dignity, cultural misinterpretations and lack of awareness contribute to inconsistent application. The quantitative data indicate a strong correlation between negative labeling and social exclusion, while qualitative insights highlight the role of religious leaders and societal norms in shaping public perception.

**Discussion:**

The study highlights Maqāṣid al-Sharı̄'ah as a potential framework to counter stigma and advocate for more inclusive policies. However, societal resistance and entrenched biases pose challenges to implementation. Addressing these issues requires enhanced public education, stronger legal enforcement, and community engagement to shift societal attitudes toward disability rights.

**Conclusion:**

This research contributes to the discourse on Islamic social justice and disability rights, emphasizing the need for a comprehensive approach to reducing stigma. Policy recommendations include involving disabled individuals in public decision-making, strengthening religious and legal discourse on inclusion, and promoting awareness campaigns to challenge societal prejudices. These efforts are essential to fostering a more equitable and inclusive society.

## Introduction

Disability is often viewed as a disease, a curse, and a social problem (Grinker, 2020; Healy, 2020; Dawn, 2023). Although every social institution guarantees justice for all members of society, social reality frequently reveals a dichotomy in the division of social groups based on impairment, leading to marginalization in social life and diversity (Jay et al., 2021).

Lessons from various countries on the fulfillment and enforcement of social justice are reflected in the laws and regulations (Svara and Brunet, 2020; Wooldridge and Bilharz, 2022), and implemented likes in Indonesia, the United States, the Netherlands, South Africa, and Australia. Indonesia upholds social justice through the 1945 Constitution (Harvelian et al., 2020), similar to other countries. The United States enforces the Civil Rights Act of 1964 (Melnick, 2024), while the Netherlands implements the *Wet gelijke behandeling* (Equal Treatment Act) of 1994 (Blom, 1995). Similarly, South Africa and Australia enforce the Constitution of the Republic of South Africa (1996) (Govender, 2016), and the Racial Discrimination Act of 1975 (Trlin, 1984), respectively, to ensure social justice without regard to background or physical condition.

In addition to the stringent laws and regulations concerning social justice known in various countries, religiously based legal frameworks offer an alternative worth considering. For instance, within Muslim communities, there is the concept of Maqāṣid al-Sharı̄'ah. This concept refers to the noble objectives of Islamic law, aimed at promoting human welfare and wellbeing (Rasool et al., 2020). It comprises five primary goals: the protection of religion, life, intellect, lineage, and property (Shihan et al., 2023). In social contexts, Maqāṣid al-Sharı̄'ah serves as a guiding principle for Muslims to balance individual rights and obligations with the welfare of the broader society (Husni et al., 2015). It emphasizes values such as compassion, justice, and tolerance as foundational in building a harmonious society. With a profound understanding of these legal objectives, Muslim communities are better positioned to approach social issues with a perspective that values both individual rights and communal wellbeing, encompassing equity, welfare, and social security.

Across international contexts, Maqāṣid al-Sharı̄'ah offers relevant guidance for addressing social issues and promoting justice within Muslim communities worldwide (Kasri et al., 2023). Its principles can be applied to evaluate existing policies or legal systems, ensuring the protection of fundamental rights while minimizing potential injustices. For example, on issues related to economic disparity or minority rights, Maqāṣid al-Sharı̄'ah provides a perspective grounded in the protection and welfare of all individuals, regardless of origin, race, or social status. Thus, Maqāṣid al-Sharı̄'ah serves not only as a moral foundation but also as a tool to drive social reform, contributing to the establishment of a fair and prosperous society.

The dynamics and demands of implementing social justice are not limited to the diversity of ethnicity, religion, race, and gender, but also include the appreciation and assurance of justice for impairment (Mladenov, 2016; Aliyu and Mustaffa, 2022). The fulfillment of the rights of disabled people is often not well-realized and faces significant challenges.

The treatment and perception of disabled individuals have been widely examined across different social and cultural contexts, often revealing substantial challenges. In Muslim-majority societies, both religious beliefs and cultural practices have been shown to either mitigate or intensify the stigma surrounding disability. Studies from countries such as Pakistan and Egypt suggest that while Islamic teachings on compassion and care for disabled individuals can reduce stigma, societal misunderstandings and misapplications of these teachings often contribute to further marginalization (Hussain et al., 2020; O'Dell, 2023). Additionally, research highlights that negative perceptions toward disabled individuals can lead to their exclusion, depriving them of their right to live fulfilling lives. Disabled Muslims, whether living in predominantly Muslim countries or as minority citizens, immigrants, or refugees in non-Muslim countries, encounter diverse challenges shaped by their unique circumstances (Ibrahim and Ismail, 2018).

Complementing these findings, Dossa (2009) examines the compounded marginalization faced by immigrant Muslim women with disabilities in Canada, illustrating how racial, gendered, and disability-based discrimination intersect to shape their experiences. Hussain (2005) similarly explores how South Asian disabled women in the UK negotiate their identities amidst patriarchy and ableism, while Turmusani (2001) delves into the impact of Islamic teachings and Middle Eastern cultural norms on disabled women's roles in society. Alnamnakani (2022) further adds to this body of research by uncovering how disabled Muslim women in the UK resist societal stigma and navigate intersecting identities. Despite the substantial contributions of these studies, a notable gap remains in research focusing on Indonesia, the largest Muslim-majority country. Indonesia's unique blend of religious, cultural, and social dynamics presents an important opportunity to explore how Islamic teachings and societal norms interact to shape the experiences and treatment of disabled individuals, particularly in urban contexts such as Makassar.

**Figure 1:**
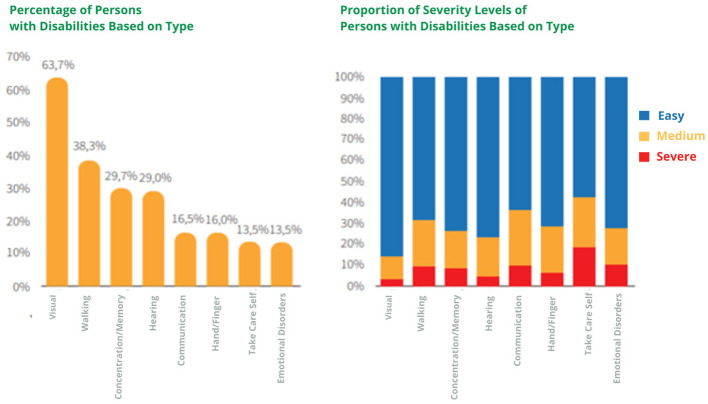
Percentage of disabled people by type and severity.

According to the 2021 report from the Central Statistics Agency (as shown in Figure 1), disabled people in Indonesia are categorized into eight groups: visual impairments, mobility impairments, memory issues, hearing impairments, communication difficulties, hand/finger impairments, self-care challenges, and emotional disturbances. The most prevalent disabilities are visual impairments, affecting 65% of the population, followed by mobility impairments at 38.3%. Despite these individuals being capable of accessing education, contributing to various employment sectors, and being regarded as equals in social status, the reality is that opportunities in education, employment, and public access remain limited, as their impairments are not perceived as severe.

**Figure 2:**
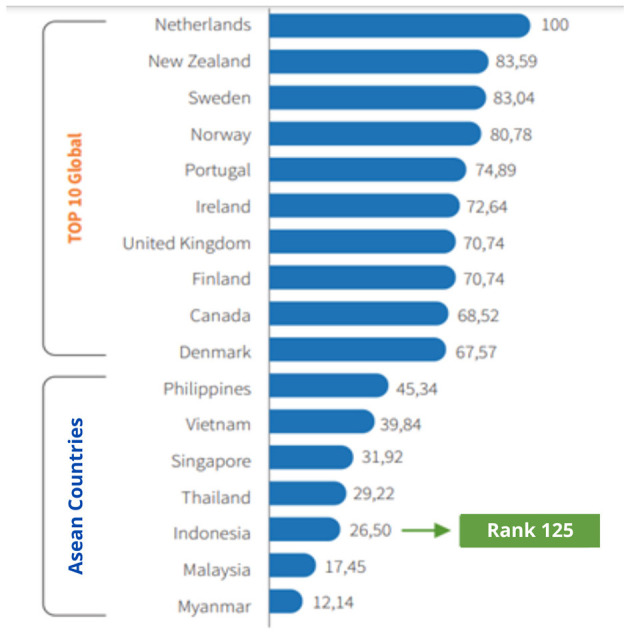
Global inclusivity index.

One of the indicators of global disability development is the inclusivity index, a holistic measure of inclusive development that focuses on gender equality, religion, race/ethnicity, and disability across various domains such as violence outside the group, incarceration rates, political representation, immigration policies, refugees, and income inequality. Unfortunately, according to statistical data, Indonesia ranks 125th with a score of 26.5% in inclusive development. This figure is lower than that of developed countries and also lags behind other ASEAN countries, as illustrated in Figure 2. This fact leads to the perception that disabled people in Indonesia have not yet received adequate services.

Various solutions must be pursued to address this issue, one of which is through religion (Sango and Forrester-Jones, 2017; Billah et al., 2023). Islam, as the majority religion in Indonesia, does not have classical scholarly discussions that specifically address disabled people. In the Qur'an and Hadith, there is no general term for disabled people, but rather the specific disabilities are mentioned, such as الأَعْمَى (the blind), as in Surah 'Abasa when the Prophet Muhammad was approached by a blind man (أَنْ جَاءَهُ الأَعْمَى) but responded with a frown. Other terms include أَنْ (the lame) and المَرِيْض (the sick) in Surah Al-Fatḥ, Chapter 48, Verse 17, as well as صُمّ (deaf) and كْم (mute) in Surah Al-Baqarah, Chapter 2, Verse 18. These terms do not always imply physical disabilities; sometimes they refer to those who refuse to see despite having normal eyesight. Similarly, in Hadith, there is no specific term for disabled people, but rather terms that refer to the specific disabilities they have.

In Arabic, the term “disabled people” is not recognized by either classical or contemporary jurists, but several general terms are used, such as أّصْحَابُ الأَعْذَار (those with excuses), which is found in several jurisprudential texts, including in al-Mabsūt by al-Ṭūsì, where a chapter is titled فَصْلُ: فِي ذِكْرِ صَلَاةِ أَصْحَابُ الأَعْذَار: مِنَ المَرِيْضِ وَالمُتَحَوِّل (Chapter: On the Prayers of Those with Excuses: Due to Illness or Distraction) (Al-Ṭūsi, 1992). Another term used is أَهْلُ البَلَاء (the people of affliction), referring to those who are tested by Allah with certain trials that prevent them from performing certain actions. Ibn Qudāmah is one of the scholars who used this term in his book al-Mugni (Al-Maqdisi, 1997).

These terms indicate that classical scholars have indeed discussed the rights of disabled people, even though a specific term for them had not been agreed upon. In contemporary terminology, several terms are used that relate to the concept of disability, such as ذُوالاِحْتِيَاجِ الخَاصَة (persons with special needs), as well as الإِعَاقَة and العَاهَة, all of which refer to individuals who have limitations in their activities, similar to what is now referred to as disability.

Despite the research conducted in other Muslim-majority regions, little has been done to explore the experiences of disabled people within Indonesia, the largest Muslim-majority country in the world. Indonesia's unique blend of religious, cultural, and social dynamics offers a critical lens through which to examine how Islamic teachings, and societal norms influence the treatment of disabled people. Although Indonesia has implemented laws and policies, such as the Law on Persons with Disabilities or disabled people, to protect the rights of disabled individuals, the lived realities of disabled Muslims in predominantly Islamic cities like Makassar remain under-researched.

Given the importance of understanding stigma in relation to both religious beliefs and societal practices, it is imperative to explore the stigma experienced by disabled Muslims in Indonesia. This study seeks to fill this gap by examining how Islamic teachings are interpreted and practiced in the daily interactions between society and disabled individuals in Makassar. Understanding these dynamics will contribute to a broader understanding of the role religion plays in shaping societal attitudes toward disability and will offer insights that may inform future policy and public education efforts aimed at reducing stigma in Islamic contexts.

The discussion is framed by the research rationale, which emphasizes the need to understand the complexities of stigma faced by disabled individuals in Makassar, the community's role in addressing such stigma, and the relevance of the Maqāṣid al-Sharı̄'ah framework in fostering inclusion and dignity. From this foundation, the study is guided by three primary research questions:

1. What are the forms of stigma faced by disabled individuals in Makassar, and how do these manifest in their daily lives? This question seeks to explore and analyze the physical, psychological, and social dimensions of stigma as experienced by the disabled community.
2. What role does the community play in addressing stigma against disabled individuals in Makassar? This question examines the contributions and challenges of families, social groups, and religious organizations in combating stigma and promoting acceptance.
3. How does the Maqāṣid al-Sharı̄'ah framework help recognize and enhance the dignity of disabled individuals based on empirical data? This question delves into the application of Islamic principles to reduce stigma and foster inclusion through legal, social, and educational measures.

## Method

This study focuses on field research, which necessitates the direct collection of data (Richard Skogley and Sawyer, 2015). The researchers gather data that is relatively unknown or has not yet been studied, utilizing the concept of applied research. This applied research concept was combined with an inductive method to derive general conclusions from the findings in the field (Guest et al., 2013). The field research was conducted directly by the researchers in Makassar to collect on-the-ground facts about cases of social discrimination, harassment, and even assault against disabled people, which have drawn public attention and made Makassar a critical area for this study.

The research process adhered to ethical considerations in collecting data from participants, ensuring their rights were respected in several keyways. Informed consent was obtained by asking participants if they were willing to participate, with no pressure to complete the questionnaire. Anonymity was maintained by not including participants names, thus protecting their privacy. Confidentiality was safeguarded to ensure that participants' information remained private. The principle of non-maleficence was upheld by providing participants with the option to agree or disagree with questions without any interference, ensuring no harm to their privacy. Finally, justice was ensured by treating all participants equally, without discrimination based on race, ethnicity, religion, or other factors.

Qualitative research is chosen to uncover these perspective, making this study primarily focused on obtaining qualitative data (Fossey et al., 2002), which will be analyzed using the concept of Maqāṣid al-Sharı̄'ah, particularly with the systems approach proposed by Auda (2008, 2011, 2016). The qualitative data gathered through semi-structured interviews were used as one of the methods of data collection, allowing flexibility while maintaining a focused line of inquiry. The interview questions were open-ended and designed to explore participants' views on disabled individuals, social interactions, and related stigma. The key participants, such as municipal government officials, activists who advocate for and support the rights of disabled people, respondents who are closely associated with stigma and religious leaders, were selected from the 400 respondents who participated in the study. We also involved disabled individuals as a informant who are at risk of facing stigma in public spaces to gain insights and perspectives from their experiences.

To support the qualitative data on stigma, the researchers employed quantitative methods as a tool to measure the level of stigma. Therefore, in this study, the researchers designed a questionnaire to identify respondents whose opinions on disabled people were sought. Quantitative methods served as supplementary data to confirm the level of stigma. The first use of quantitative data was in measuring stigma against disabled people in Makassar, using a Likert scale questionnaire (Joshi et al., 2015).

Participants included individuals in Makassar, Indonesia, who interact with disabled people, such as family members, neighbors, and service providers. A simple random sampling method was used to ensure broad representation from the general population, with the sample size calculated using the Taro Yamane formula (Yamane, 1967). The study aimed to gather data from 400 respondents, a number determined to be statistically sufficient to represent the population (File, Population, and Sample section). Of these, 200 respondents identified as Muslim, while the remaining 200 chose not to disclose their religious background.

Inclusion criteria required participants to have interacted with disabled individuals in their daily lives, either personally or professionally, without restrictions based on age or gender. Exclusion criteria consisted of individuals who refused to provide consent to participate in the study.

## Results

### Analysis of respondents' characteristics and interactions with disabled people

The stigma experienced by disabled people in Makassar is based on data processed from 400 respondents, with the assumption that all samples have observed and interacted with disabled people across different age groups, from children to adults and the elderly. The data is categorized by gender, age group, and type of interaction, examining key stigma-related terms such as harassment stigma, insult stigma, and negative labeling of disabled people.

This study focuses on individuals who interact with disabled people. The decision to focus on individuals who interact with disabled people, rather than the disabled themselves, was made to capture societal perspectives and behaviors that contribute to stigma. By studying those who interact with disabled people, such as family members, colleagues, or service providers, we can gain insight into how societal attitudes and biases are manifested. These individuals often play a role in shaping the environment and experiences that disabled people encounter daily, whether through conscious or unconscious behavior. Their perspectives can reveal how stigma is perpetuated or challenged in various social settings, as well as how Islamic law responds to such situations.

Moreover, studying the perspectives of non-disabled individuals helps highlight the social barriers—such as discrimination, exclusion, or misinformation—that disabled people face, which may not always be apparent when focusing solely on the reports of disabled people themselves. This external viewpoint is crucial for understanding the systemic and cultural sources of stigma affecting disabled people. Therefore, the responses from those who interact with disabled people provide valuable insights into the broader social dynamics that contribute to the stigma experienced by disabled people.

**Figure 3:**
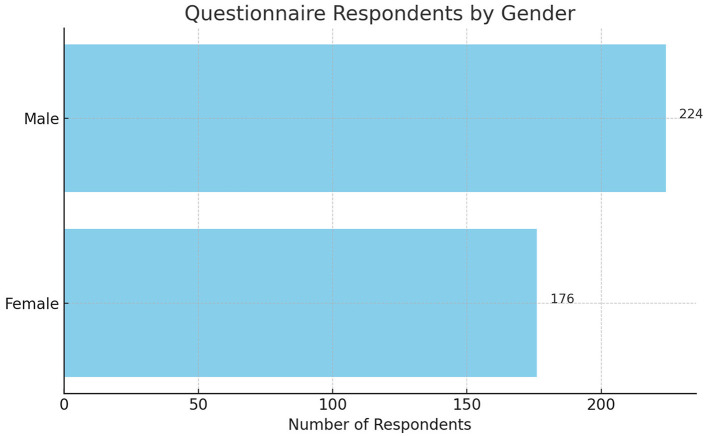
Distribution of respondents' gender.

The questionnaire distributed by the researchers is categorized from several aspects, the first of which is gender. Based on the Figure 3, the majority of respondents who were willing to complete the questionnaire were male, numbering 224, while 176 were female.

The next aspect is age group. According to basic health research data, the age groups of disabled people can be categorized into three categories: children with disabilities, adults with disabilities, and elderly disabled people.

**Figure 4:**
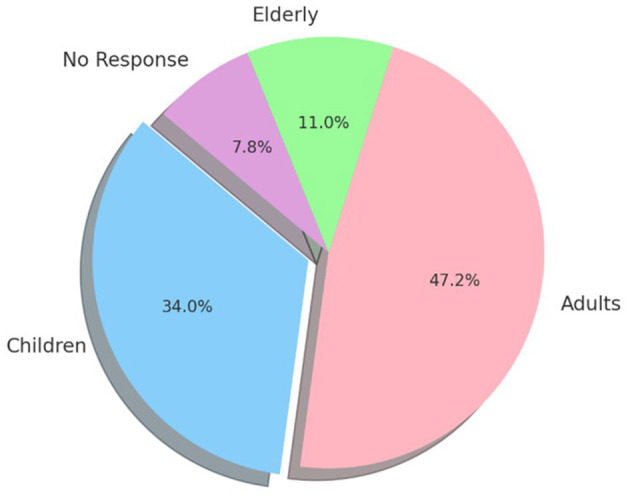
Age group distribution of respondents' interactions with disabled people.

Figure 4 shows that respondents interacted most frequently with adults with disabilities, aged between 18 and 60 years (189 individuals), followed by children with disabilities (136 individuals). The least interaction was with elderly disabled individuals, aged 60 years and above (44 individuals), while 31 respondents did not provide an answer. This data provides researchers with the opportunity to understand the interaction process between society and disabled people who are in their productive years, focusing on their ability to work and obtain rights in public spaces.

The final aspect is the form of interaction. The type of interaction between respondents and disabled people is one of the factors measured by the researchers to understand the nature of these interactions.

**Figure 5:**
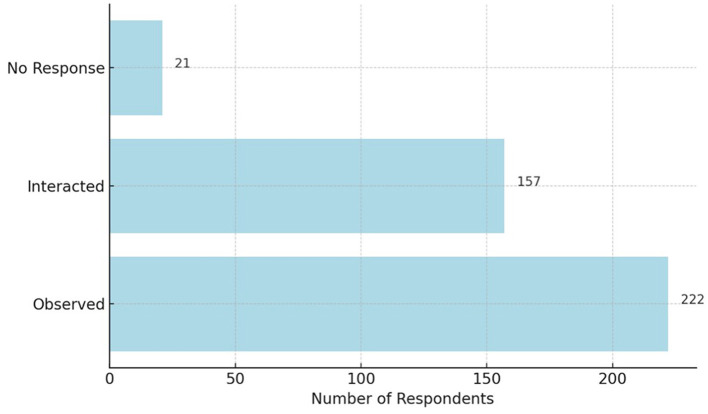
Form of interaction with disabled people.

Based on Figure 5, it is evident that most respondents only observed the condition of disabled people (222 individuals), although the number of respondents who directly interacted with disabled people is also significant (157 individuals), with 21 respondents not providing an answer. This finding serves as an initial argument for the high levels of stigma against disabled people in Makassar, as one of the indicators of stigma is the presence of mere perception.

### How is the harassment and stigmatization of disabled people?

Stigma-related expressions such as harassment, insults, and negative labeling directed at disabled people. Harassment stigma can manifest against disabled people in the form of physical and psychological abuse, both verbal and non-verbal. The findings of this study show that respondents perceive disabled people as being physically different from themselves.

The perception of respondents in Table 1, who consider themselves physically different from disabled people, is categorized as very high. The strong perception among respondents that they are physically different from disabled people can lead to stigma, which may result in harassment. The physical differences and limitations experienced by disabled people can create opportunities for those who feel they can exploit these conditions.

“People's pity makes us look different. But we are just as capable of doing our work as anyone else. Excessive pity can even lead to new forms of harassment and stigma against us.” (Informant 2—Disabled Person)

“I doubt they can do as well as others. Maybe it would be better if they were given less strenuous work. He is not capable of heavy work.” (Participant 159)

“I prefer to hire people without physical limitations. They would only slow down productivity. In a competitive work environment, their presence stands out significantly.” (Participant 87)

**Table 1:** Respondents on physical differences in disabilities.

**Response**	**Number (*T* × *C*)**	**Percentage (Total score/*Y* × 100)**	**Result**
Strongly disagree	16	77.1875%	Very high
Disagree	108		
Agree	567		
Strongly agree	544		
Total score	1,235		

The societal stigma regarding the burdensome nature of disabled people due to their condition is relatively high, as shown in Table 2. This indicates that there is a stigma suggesting that the physical form of disabled people is perceived negatively, which can lead to insults and even harassment.

**Table 2:** Respondents on disabilities feeling burdened.

**Response**	**Number (*T* × *C*)**	**Percentage (Total score/*Y* × 100)**	**Result**
Strongly disagree	40	62%	High
Disagree	312		
Agree	522		
Strongly agree	116		
Total score	990		

The high level of societal stigma toward disabled people, which can lead to harassment, becomes evident when researchers explore the various forms of harassment, such as physical abuse.

Although Table 3 shows a significant number of respondents who disagree with the vulnerability of disabled people to being touched, the number of respondents who agree with this view is also considerable. This contributes to the high stigma of harassment against disabled people and suggests that the likelihood of harassment occurring in Makassar is relatively high. Additionally, respondents also believe that disabled people are at risk of being subjected to inappropriate behavior by those around them.

“Because of their physical limitations, disabled people are vulnerable to ridicule. This perception is shaped by societal norms that view disability as a curse or merely as content for entertainment.” (Participant 212)

“The lack of convenient services for disabled individuals provided by the Makassar City Government in public facilities highlights the significant disparity in accessibility.” (Participant 345)

**Table 3:** Respondents on disability and physical vulnerability.

**Response**	**Number (*T* × *C*)**	**Percentage (Total score/*Y* × 100)**	**Result**
Strongly disagree	115	53%	High
Disagree	290		
Agree	372		
Strongly agree	64		
Total score	841		

According to respondents in Table 4, disabled people have a high likelihood of being subjected to inappropriate behavior due to the strong stigma against them. This clearly demonstrates the significant stigma directed toward disabled people, particularly in terms of harassment, with respondents believing that the likelihood of sexual harassment against disabled people is much higher.

“The factors that lead to harassment of disabled people can stem from various causes, such as families feeling ashamed of having a child with a disability or families being unable to protect their disabled relatives. This is a reality we often hear as advocates.” (Participant 22)

“Building a harmonious family is key to ensuring that families can protect their members with disabilities.” (Participant 56)

“Environments prone to harassment, such as slum areas, lack of public spaces for disabled people, weak law enforcement, and lack of community oversight, all contribute to the high risk of harassment against disabled people. My friends and I have personally experienced this.” (Informant 1—Disabled Person)

**Table 4:** Respondents on disability vulnerability to inappropriate behavior.

**Response**	**Number (*T* × *C*)**	**Percentage (Total score/*Y* × 100)**	**Result**
Strongly disagree	139	55%	High
Disagree	158		
Agree	423		
Strongly agree	160		
Total score	880		

However, the high stigma regarding the likelihood of harassment contrasts with the responses given when researchers asked respondents to report incidents of harassment they had personally witnessed.

Respondents to the question reflected in Table 5 tend to report that the incidence of sexual harassment they have personally witnessed involving disabled people is relatively low. However, despite the low numbers, there is still a significant portion of respondents who agree with this statement.

**Table 5:** Respondents on witnessing forced kissing, rape, or exploitation of disabled people.

**Response**	**Number (*T* × *C*)**	**Percentage (Total score/*Y* × 100)**	**Result**
Strongly disagree	244	39%	Low
Disagree	194		
Agree	126		
Strongly agree	64		
Total score	628		

Based on the data presented, it is evident that societal stigma in Makassar toward disabled people remains relatively high, particularly in terms of harassment. As a result, disabled people are always at risk of experiencing physical, psychological, verbal, and non-verbal harassment.

“I rarely come across cases of witnessing forced kissing, rape, or exploitation of disabled people firsthand. However, I have read about such incidents several times in local online media.” (Participant 122)

Therefore, there is a need for solutions that primarily focus on the wellbeing of disabled people, including the following: First, sexual education specifically for disabled people. Although it may be considered taboo, it is necessary for disabled people to understand which parts of their bodies can and cannot be touched by others, as well as to receive education on reproductive health supported by scientific data. Second, raising awareness about the prevention of sexual violence against disabled people. They should be taught about the dangers and consequences of harassment, particularly toward disabled people. Third, intervention by paying attention to events, perceiving situations that require immediate help, being responsible for addressing incidents, knowing how to act and report, so that disabled people should not remain silent in the face of harassment. They must actively participate if they suspect that harassment is about to occur.

### Insults as stigma toward disabled people

Insult is a process, method, or act of degrading or defaming someone (Daly, 2018). It can also be understood as a criminal act and defamation, which can take various forms, including (a) oral defamation/verbal slander; (b) written defamation/libel; (c) slander; (d) minor insult; (e) false accusation; (f) creating false suspicion; (g) insult regarding a deceased person. The causes of insult generally stem from ignorance about the object of the insult, as well as emotional factors that lead to the victim being insulted.

The results of the questionnaire in Table 6 show that the majority of respondents agree that disabled people were found to be highly vulnerable to being insulted. This, of course, had a significant impact on the potential threat to their safety in public places, as it can lead to discomfort and even discrimination against them.

“We frequently encounter bullying against students with dwarfism in schools. Words such as ‘**dattulu**,' ‘**tuyul**,' and ‘**cebol**' are often heard.” (Participant 366)

**Table 6:** Respondents on vulnerability of disabled people to insults.

**Response**	**Number (T × C)**	**Percentage (Total score/*Y* × 100)**	**Result**
Strongly disagree	127	56%	High
Disagree	164		
Agree	393		
Strongly agree	216		
Total score	900		

This is evident from an incident that occurred in Makassar, where a 13-year-old student with dwarfism was frequently bullied by classmates over the past month. However, the situation only came to public attention after a video of the student's head being kicked went viral, causing severe trauma to the student and their family. This reality underscores that insults against disabled people in Makassar are both real and prevalent.

In a different dimension of questioning, the findings related to insults directly witnessed by respondents are relatively low. Respondents indicated that they have never insulted disabled people or their families.

The findings from respondents in Table 7 provided an opportunity to prevent the stigma of insults against disabled people through welfare-based prevention measures. One such prevention strategy can be the enhancement of protection for disabled people from individuals who hold the stigma that they are vulnerable to insults.

“There is a significant difference between disabled individuals from poor and wealthy families. The stigma and perceptions are different. In lower-income communities, wealthy individuals are highly respected, even if they have disabilities.” (Participant 187)

“In our culture, people are respected based on social status, education, and wealth. There is almost no stigma or discrimination against these three aspects.” (Participant 111)

**Table 7:** Respondents on insults directed by them at disabled people.

**Response**	**Number (*T* × *C*)**	**Percentage (Total score/*Y* × 100)**	**Result**
Strongly disagree	263	36%	Low
Disagree	180		
Agree	117		
Strongly agree	20		
Total score	580		

From the perspective of Maqāṣid al-Sharı̄'ah, preventing insults against disabled people must be based on fundamental principles that protect human dignity and honor. Maqāṣid al-Sharı̄'ah, which focuses on the protection of five basic things—religion, life, intellect, lineage, and property—provides a strong framework for understanding and preventing such insults. Insulting disabled people is a violation of the principles of protecting life and human dignity. In Islam, every individual, regardless of their physical or mental condition, possesses dignity that must be respected. Overall, the perspective of Maqāṣid al-Sharı̄'ah demands a holistic approach to preventing insults against disabled people, focusing on education, law, inclusion, empowerment, and social support, to ensure that all individuals can live with the dignity and respect they deserve.

The low results reflected in Table 8 provide an opportunity to offer education aimed at reducing the high stigma that disabled people are prone to insults, as respondents do not feel disgusted by the condition of disabled people.

**Table 8:** Respondents' reactions to physical forms of disabilities that elicit disgust.

**Response**	**Number (*T* × *C*)**	**Percentage (Total score/*Y* × 100)**	**Result**
Strongly disagree	265	36%	Low
Disagree	208		
Agree	54		
Strongly agree	48		
Total score	575		

The greatest support that can help disabled people avoid insults is the presence of family. Research conducted by Liu Fengbo has shown that the presence of family provides social support and happiness to disabled people, and the family's role can even alleviate various social problems, as the family can act as a mediator in case of issues (Fengbo et al., 2024). However, in the findings gathered by the researchers, many respondents believe that disabled people receive insufficient attention from their families.

Maqāṣid al-Sharı̄'ah emphasizes the importance of treating all individuals with respect and compassion, in accordance with the teaching that humans are creations of Allah who must be valued and protected. Additionally, the view that disabled people are repulsive can harm their mental and emotional wellbeing. This contradicts the principles of Maqāṣid al-Sharı̄'ah, which seek to protect intellect and mental health. Discriminatory and demeaning treatment can cause psychological trauma and worsen the mental health conditions of disabled people. Islam teaches the importance of creating a loving and supportive environment for all family members, including those with disabilities. From the perspective of protecting lineage and family, negative views toward disabled people can undermine family harmony and unity. Maqāṣid al-Sharı̄'ah emphasizes the importance of maintaining family honor and stability. Discrimination against family members with disabilities can lead to tension and disharmony within the family, which goes against Islam's goal of creating a loving and supportive family environment. Degrading views also conflict with the principles of justice and social welfare in Maqāṣid al-Sharı̄'ah. Social justice in Islam includes fair and inclusive treatment for all individuals, including disabled people, and rejects all forms of discrimination. Considering disabled people as repulsive is a form of injustice that disregards their rights to be accepted and treated equally in society.

Table 9 illustrates the high level of respondent agreement regarding the lack of family attention toward disabled people. According to the Convention on the Rights of the Child, every child has the right to survival, protection, development, and participation. The lack of family attention to disabled people significantly increases the likelihood of escalating stigma, particularly leading to harassment. Therefore, enhancing the role of the family should be prioritized to ensure greater attention is given to children with disabilities.

**Table 9:** Respondents on lack of family attention to disabled people.

**Response**	**Number (*T* × *C*)**	**Percentage (Total score/*Y* × 100)**	**Result**
Strongly disagree	102	53%	High
Disagree	320		
Agree	348		
Strongly agree	84		
Total score	854		

The data in Table 10 indicates that there is a belief that families should not need to hide the fact that they have a child with a disability. However, in reality, Dante Rigmalia (Chairperson of the National Disability Commission) states that the stigma against children with disabilities remains very strong. This is evident from the widespread bullying and arbitrary rejection by society, and even by families. This is largely due to the lack of knowledge among families and parents about children with disabilities, leading to children with disabilities being abandoned by their families and sent to social institutions.

**Table 10:** Respondents on the view that disabilities are a family disgrace.

**Response**	**Number (*T* × *C*)**	**Percentage (Total score/*Y* × 100)**	**Result**
Strongly disagree	244	38.3125%	Low
Disagree	230		
Agree	75		
Strongly agree	64		
Total score	613		

In Indonesia, the perception that disability is a family disgrace is deeply rooted in cultural, social, and beliefs (Kiling et al., 2019; Subu et al., 2023). Many communities associate disability with negative connotations (Kusumastuti et al., 2014), viewing it as a result of past misdeeds or divine punishment. This belief is reinforced by cultural norms that emphasize familial reputation and social status, particularly in rural areas where societal acceptance is heavily influenced by collective identity. As a result, families with disabled members may experience social exclusion, as the presence of disability is often seen as a failure to conform to societal expectations of health, productivity, and normalcy.

“At first, my family struggled to accept my situation as a disabled person. However, over time, with clear religious guidance on gratitude, mutual respect, and support, they eventually accepted me, making it feel more normal.” (Informant 3—Disabled Person)

In some cases, misinterpretations of religious teachings lead to the belief that disability is a trial or test from God, further stigmatizing the individual and the family. The lack of public awareness and education about disability exacerbates this stigma, as families may internalize these beliefs, feeling shame or guilt for having a disabled family member.

This perception is further perpetuated by the limited visibility of disabled individuals in public spaces, as families may choose to hide or isolate disabled members to avoid judgment or gossip. Even more tragically, some families resort to imprisoning disabled members in inhumane ways, a practice known as *Pasung* (Baklien et al., 2023). The absence of adequate government support and the scarcity of accessible public services contribute to this marginalization, reinforcing the idea that disability is a private issue rather than a societal one.

Considering disabled people as a disgrace contradicts the principles of protecting human life and dignity. Islam teaches that every individual, regardless of their physical or mental condition, possesses dignity that must be respected. Viewing disabled people as a disgrace devalues their dignity and fails to recognize the human values instilled in Islamic teachings. Maqāṣid al-Sharı̄'ah emphasizes the importance of treating all individuals with respect and compassion, in line with the belief that humans are creations of Allah who must be honored and protected.

Additionally, the stigma of being considered a disgrace can harm the mental and emotional wellbeing of disabled people. This is contrary to the principles of Maqāṣid al-Sharı̄'ah, which seek to protect intellect and mental health. Any form of discrimination or exclusion can cause psychological trauma and worsen their mental health conditions. Islam teaches the importance of mutual support and providing a loving and understanding environment for all family members, including those with disabilities.

From the perspective of protecting lineage and family, considering disabled people as a disgrace can undermine family harmony and unity. Maqāṣid al-Sharı̄'ah emphasizes the importance of maintaining family honor and stability. Discrimination against family members with disabilities can lead to tension and disharmony within the family, which goes against Islam's goal of creating a loving and supportive family environment.

### Societal dynamics in the negative labeling of disabled people

Labeling (stereotyping) is the generalization of individuals within a group without sufficient information, while ignoring the characteristics of the individuals in that group (Gershman and Cikara, 2023). Stereotypes are formed through various means, such as personal experiences, relevant experiences of others, and media influence. Stereotypes about disabled people develop within social categories and can be clearly categorized because disability is perceived as something unchangeable, like ethnicity, race, or sex (Wicaksono et al., 2021). Respondents believed that disabled people still receive negative stereotypes from society.

“Disabled people are sometimes perceived as insane, possessed, or as a punishment from God. In reality, the root cause often lies in the lack of access to proper nutrition during pregnancy.” (Participant 342)

The high stigma against disabled people, coupled with negative stereotypes as shown in Table 11, positions disabilities as a disease. Even more concerning, disabled people are often denied the inclusive rights to participate in various aspects of life, particularly those related to social issues. In reality, disabled people are like anyone else—sometimes healthy and sometimes ill. This negative labeling also occurs in Makassar.

“It is not uncommon for disabled people to face rejection due to the perception that they are burdensome, when in fact, this is not the case. For example, there is a common misconception that restrooms for disabled individuals must always be specially designed. However, in reality, not all disabled workers require specialized facilities.” (Participant 44)

**Table 11:** Respondents' views on negative stereotypes toward disabilities.

**Response**	**Number (*T* × *C*)**	**Percentage (Total score/*Y* × 100)**	**Result**
Strongly disagree	58	67.188%	High
Disagree	176		
Agree	525		
Strongly agree	316		
Total score	1,075		

Using negative labels against disabled people can degrade their dignity and disregard the human values emphasized in Islamic teachings. Maqāṣid al-Sharı̄'ah demands that all individuals be treated with justice and compassion, in accordance with the belief that humans are creations of Allah who must be respected. Furthermore, negative views toward disabled people can harm their mental and emotional wellbeing. This contradicts the principles of Maqāṣid al-Sharı̄'ah, which aim to protect intellect and mental health. Discriminatory treatment or exclusion can lead to psychological trauma and worsen their health conditions. Islam teaches the importance of creating a compassionate and supportive environment for all members of society, including disabled people.

From the perspective of family and lineage protection, negative views toward disabled people can undermine family harmony and unity. Maqāṣid al-Sharı̄'ah emphasizes the importance of maintaining family honor and stability. Discrimination against family members with disabilities can create tension and disharmony within family relationships, which contradicts Islam's goal of fostering a loving and supportive family environment. Moreover, negative views toward disabled people can violate the principles of justice and social welfare. Social justice in Islam involves fair and inclusive treatment of all individuals, regardless of their physical or mental condition. Using negative labels against disabled people is a form of injustice that disregards their rights to be accepted and treated equally in society.

In matters related to societal stigma regarding employment opportunities, respondents view disabled people differently. The high societal stigma regarding the lack of employment opportunities for disabled people, as shown in Table 12, further reinforces negative labeling against them. In reality, disabled people have the same and equal rights to access employment in order to meet their livelihood needs. The state is obligated to protect, fulfill, and enforce the rights applicable to all citizens, regardless of their status, including disabilities. Several strategies that the government can implement to uphold the rights of disabled people in relation to employment include providing special treatment for them, especially in terms of inclusive rights. However, in practice, it remains challenging to enforce rules on employing disabled people due to difficulties in finding suitable job materials that meet employers' demands, inconsistent implementation of the 1% quota for disabled people, low awareness of the importance of employing disabled people, and the lack of motivation among disabled people to seek employment.

**Table 12:** Respondents on the perception that disabled people cannot work.

**Response**	**Number (*T* × *C*)**	**Percentage (Total score/*Y* × 100)**	**Result**
Strongly disagree	127	51%	High
Disagree	314		
Agree	246		
Strongly agree	128		
Total score	815		

The view that disabled people are unfit to work can also undermine the human values in Islam. Maqāṣid al-Sharı̄'ah emphasizes the importance of respecting and protecting the dignity of every individual. Considering them unfit to work diminishes their dignity and hinders their efforts to achieve economic and social independence. From the perspective of general welfare and social prosperity, Maqāṣid al-Sharı̄'ah highlights the importance of building an inclusive and just society. Providing equal employment opportunities to all individuals, including disabled people, is part of the effort to achieve the social balance and wellbeing desired in Islam. This aligns with the principle of social justice, which emphasizes fair treatment and equality, advocating for equal rights for all members of society.

Respondents show positive support for the need to enhance individual development and acceptance within the social environment, a shown in Table 13. Special treatment for disabled people is guaranteed under Article 28H paragraph (2) of the 1945 Constitution, which affirms that everyone has the right to receive assistance and special treatment to obtain equal opportunities and benefits in order to achieve equality and justice. Therefore, to reduce the high stigma surrounding employment opportunities for disabled people, it is necessary to seek solutions based on public welfare.

**Table 13:** Respondents on special treatment for disabled people in social and individual development.

**Response**	**Number (*T* × *C*)**	**Percentage (Total score/*Y* × 100)**	**Result**
Strongly disagree	14	86.063%	Very high
Disagree	38		
Agree	429		
Strongly agree	896		
Total score	1,377		

Disabled people have the right to special protection because Islam teaches that every individual, regardless of their physical or mental condition, possesses dignity that must be respected and protected.

This view is reflected in the principles of Maqāṣid al-Sharı̄'ah, which outline the primary objectives of Islamic law to preserve human life and society in a just and balanced manner. First and foremost, special protection for disabled people is necessary to safeguard their dignity and wellbeing. Islam individual is created by Allah with a specific purpose and uniqueness. Ignoring or sidelining their needs means failing to respect the human values taught in Islamic teachings. Maqāṣid al-Sharı̄'ah demands that every individual, including disabled people, be treated with compassion and understanding.

Additionally, special protection is necessary to ensure that disabled people have equal access to the services and opportunities they need. This includes access to education, healthcare, transportation, employment, and various other social activities. Maqāṣid al-Sharı̄'ah emphasizes the importance of social justice and inclusion, where all individuals have the same rights to participate actively in society. From the perspective of family and lineage protection, Maqāṣid al-Sharı̄'ah places significant responsibility on the family to protect its vulnerable members, including disabled people. This includes providing care, support, and an environment that enables them to develop to their fullest potential. A well-functioning family, according to Islamic teachings, is one that nurtures the harmony and wellbeing of all its members, regardless of their condition. More broadly, special protection for disabled people reflects Islam's commitment to justice and humanity. Islam teaches that every individual should be treated fairly and equally in all matters, without discrimination based on status or physical condition. Providing special protection for disabled people is an integral part of creating an inclusive and just society, in line with Islam's vision of general welfare and social harmony.

In terms of assumptions regarding access to education, respondents believe that disabled people should have the same opportunities to attend school. Respondents in Table 14 believe that disabled people have the right to an education, which aligns with the availability of Special Schools in Makassar, although the total number is still relatively small-−2 State Special Schools and 20 Private Special Schools. Therefore, parents should not neglect the educational rights that children with disabilities are entitled to receive.

“In accordance with the mandate of the law, the Makassar city government prepares special school and supports private special schools in creating a high-quality learning process for students with disabilities.” (Participant 279)

“We, from the Yayasan Pembinaan Tunanetra Indonesia (YAPTI), provide access to religious education for disabled people, particularly those who are visually impaired, through our religious learning programs, including the Braille Qur'an recitation program. The use of the Braille Qur'an requires blind individuals to first learn the alphabet, as the Braille Qur'an is composed of alphabetic letters.” (Participant 338)

**Table 14:** Respondents on disabled people unable to attend school.

**Response**	**Number (*T* × *C*)**	**Percentage (Total score/*Y* × 100)**	**Result**
Strongly disagree	188	44.1875%	Low
Disagree	278		
Agree	141		
Strongly agree	100		
Total score	707		

Although the desire for education is relatively high, disabled people still need a lot of encouragement to pursue education. According to the 2018 Rikesda data, 22% of disabled people were of school age, but as of that time, only 30.7% had completed secondary education. The impact of inadequate educational programs for disabled people is a reduced opportunity to obtain suitable employment, which may contribute to the increase in societal stigma.

Islam teaches that every individual, including disabled people, has the same right to develop their potential through education. Education is not only seen as a means to acquire knowledge but also as a way to build individual dignity and enable them to contribute meaningfully to society. In this context, Maqāṣid al-Sharı̄'ah emphasizes the importance of eliminating all forms of discrimination and providing equal access to education for everyone. Islam encourages comprehensive social inclusion. Providing adequate education to disabled people helps create a more just society. Those who receive a good education have a greater opportunity to achieve economic independence, improve their quality of life, and contribute to the overall development of society. In this regard, Maqāṣid al-Sharı̄'ah highlights that education is a long-term investment in the prosperity and wellbeing of humanity.

Society believes that disabled people have the right to access education, as shown in Table 15. However, this view contradicts their perception of employment opportunities for disabled people. As previously explained, disabled people face significant challenges in accessing employment due to a variety of factors.

“Although access to employment is limited, disabled people still need to be given opportunities to work. In Makassar, I have seen several small businesses, micro-enterprises, and cooperatives, such as Café Tulus and Tenoon.id, that involve disabled people as workers.” (Participant 183)

**Table 15:** Respondents on disabled people unable to work.

**Response**	**Number (*T* × *C*)**	**Percentage (Total score/*Y* × 100)**	**Result**
Strongly disagree	127	51%	High
Disagree	314		
Agree	246		
Strongly agree	128		
Total score	815		

Respondents also believe that disabled people still need assistance in decision-making, as shown in Table 16. This stigma leads to the labeling of disabled people as dependent and unable to make decisions on their own, as professionals and parents often fear that they will fail. This perception also contributes to the lack of skill development among disabled people.

**Table 16:** Respondents on disabled people need assistance in decision-making.

**Response**	**Number (*T* × *C*)**	**Percentage (Total score/*Y* × 100)**	**Result**
Strongly disagree	24	71.563%	Very High
Disagree	172		
Agree	597		
Strongly agree	352		
Total score	1,145		

Maqāṣid al-Sharı̄'ah demands that every individual, including disabled people, have equal access to the decision-making processes that affect their lives. This includes their right to be involved in decisions related to health, finances, education, and other important aspects of daily life. In this context, society is encouraged to provide a supportive environment where disabled people can actively participate in the decision-making processes that impact them directly.

In matters of marriage, disabled people still face significant challenges in Makassar. Based on the data in Table 17, respondents continue to hold the stigma of not marrying disabled people.

**Table 17:** Respondents' reluctance to marry into a family with disabilities.

**Response**	**Number (*T* × *C*)**	**Percentage (Total score/*Y* × 100)**	**Result**
Strongly disagree	102	54.375%	High
Disagree	308		
Agree	348		
Strongly agree	112		
Total score	870		

The high societal stigma against marrying disabled people indicates a strong negative label attached to them, as they are perceived as being unprepared to manage a household. However, even though respondents may not want to marry disabled people, they still believe that disabled people have the right to marry and become heads of families.

Based on the data in Table 17, respondents are unwilling to consider disabled people as their life partners. However, research shows that living together, including marriage between persons with and without disabilities, can remain long-lasting and harmonious with effective relationship maintenance strategies.

“There is still a fear that marrying a disabled person might result in our children inheriting the same disability.” (Participant 31)

“Alhamdulillah, my husband and family fully accept my condition as it is because from the beginning, there was commitment and acceptance.” (Informant 4—Disabled Person)

Maqāṣid al-Sharı̄'ah emphasizes the importance of not judging a person's ability to marry based on their disability, as marriage is a fundamental human right regardless of any disabilities they may have (Bakry, 2019). Instead, the focus should be on their ability to fulfill the responsibilities as a spouse and family member. Treating disabled people fairly and respecting their rights to lead a dignified family life is an implementation of Islamic values that advocate for justice, equality, and compassion in social relationships.

Although negative labeling stigmas remain relatively high, respondents are willing to accept disabled people as friends, as shown in Table 18.

“I am friends with disabled people. There is no difference because we are all creations of God. Helping and supporting each other is far more important.” (Participant 41)

**Table 18:** Respondents' reluctance to associate with disabled people.

**Response**	**Number (*T* × *C*)**	**Percentage (Total score/*Y* × 100)**	**Result**
Strongly disagree	252	36.5%	Low
Disagree	242		
Agree	42		
Strongly agree	48		
Total score	584		

After discussing the three main points of protecting disabled people against stigma, particularly in terms of harassment, insults, and negative labeling related to their disability, it is clear that the level of stigma in Makassar remains high. Therefore, the findings suggest various strategic steps to reduce stigma against disabled people in Makassar. Key points for reducing this stigma include the importance of socialization and education for the public about human rights based on tangible benefits, increasing mutual awareness between persons with and without disabilities, and enhancing the knowledge of the people of Makassar using the foundation of Maqāṣid al-Sharı̄'ah concerning disabled people.

## Discussion and implications

The term “persons with disabilities” is an adaptation of the terminology established by the Convention on the Rights of Persons with Disabilities (CRPD) (Nurhaeni et al., 2024). The Convention provides a new foundation for understanding and recognizing the principles of protection, respect, and fulfillment of the rights of disabled people in various countries to this day. In other terminology, “disabled people” is also used, emphasizing that individuals are disabled by societal barriers, such as stigma, rather than by their bodies (Shakespeare, 2013).

In Makassar, a city with a diverse demographic and cultural background, disabled people face significant stigma, which is deeply embedded in social interactions and perceptions. This stigma manifests in various forms, including physical, psychological, verbal, and non-verbal abuses, often leading to the marginalization of individuals with disabilities. The findings from this study, grounded in the Maqāṣid al-Sharı̄'ah perspective, shed light on the complexities of these stigmas and offer a framework for understanding and addressing them within an Islamic context.

Maqāṣid al-Sharı̄'ah and the protection of the rights of persons with disabilities or disabled people have a strong relationship within Islam. Maqāṣid al-Sharı̄'ah refers to the objectives underlying Islamic law. The term Maqāṣid (مَقَاصِد) is the plural form of maqṣid (قَصَدَ) and is derived from the Arabic root qaṣada (قَصَدَ), meaning “purpose” or “objective,” while Shari‘ah (الشَّرِيْعَة) comes from the root shara'a (شرع), meaning “law” (Wafa, 2021). In terminology, Maqāṣid al-Sharı̄'ah is defined as (al-Fasi, 1963):

الغَايَةُ مِنْهَا وَالأَسْرَارُ الَّتِيْ وَضَعَهَا الشَّارِعُ عِنْدَ كُلِّ حُكْمٍ مِنْ أَحْكَامِهَا.

“The objectives and wisdom behind the laws (Sharı̄'ah) that have been established by the lawgiver (al-Shāri‘) in every ruling of its laws.”

Thus, broadly speaking, every law established by Allah (SWT) and the Prophet Muhammad carries inherent wisdom, which can be categorized into three essential principles: Ḍaruriyyāt (necessities), Ḍājiyyāt (needs), and Taḥsiniyyāt (complementary or embellishments).

These concepts—Ḍaruriyyāt, ḥājiyyāt, and Taḥsiniyyāt—relate to the hierarchy of needs in Islam and are highly relevant to understanding the rights of disabled people. Islam emphasizes the protection and fulfillment of the needs of disabled individuals. They have the same rights to meet their Ḍaruriyyāt (basic needs), Ḍājiyyāt (essential needs), and Taḥsiniyyāt (higher complementary needs). The following sections will explain the definitions and relationships of Ḍaruriyyāt, Ḍājiyyāt, and Taḥsiniyyāt with disabled people, along with the support found in the Qur'an and Hadith, all provided in Arabic.

The progressive understanding of Maqāṣid al-Sharı̄'ah has evolved, increasingly focusing on empowerment after centuries of being one of the key theories in resolving issues in Islamic law. Jasser Auda, an Egyptian scholar, argues that Maqāṣid al-Sharı̄'ah represents the intentions and objectives of the Lawgiver (Allah SWT and the Prophet Muhammad), realized in the form of tasyri' (legislation) through rulings established by mujtahids via the process of istinbat (extraction) from the sacred texts of Sharı̄'ah (Auda, 2008, 2011, 2016). According to Auda, the components of Maqāṣid al-Sharı̄'ah are closely tied to the values that the Lawgiver seeks to realize, which are then investigated by mujtahids through Sharı̄'ah texts (revelation) (Auda, 2008, 2011, 2016).

Auda expands Maqāṣid al-Sharı̄'ah into a broader framework, emphasizing four key aspects (Auda, 2008, 2011, 2016). First, he categorizes Maqāṣid al-Sharı̄'ah into three levels, beginning with maqāṣid al-'āmmah, which encompasses universal wellbeing through principles such as equal rights, justice, tolerance, and obligations. These general objectives aim to ensure contemporary relevance by promoting development (al-Tanmiyyah) in religion, life, intellect, lineage, and wealth. Second, he extends Maqāṣid al-Sharı̄'ah beyond individual protection to encompass the development of societies, nations, and states. Third, the sources of Maqāṣid al-Sharı̄'ah are directly derived by mujtahids from the Qur'an and Hadith. Fourth, its objectives are not confined to protection and preservation but also emphasize human development, human rights, and comprehensive welfare.

**Figure 6:**
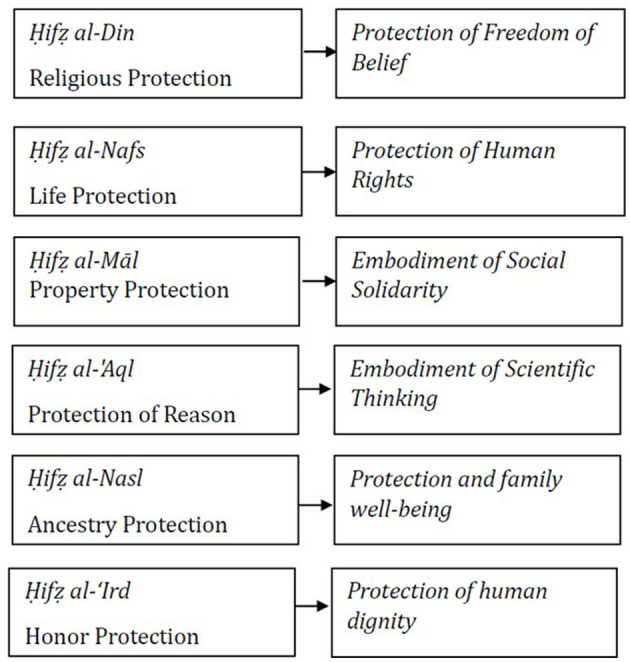
The scope of Maqāṣid al-Sharı̄'ah by Jasser Auda.

Auda also introduces a systems approach (Auda, 2008, 2011, 2016), which underscores the interconnectivity of Maqāṣid al-Sharı̄'ah and the necessity of expanding its scope to encompass social and public dimensions. Rather than focusing solely on individual protection, Maqāṣid should extend to the wellbeing, development, and empowerment of society. This broader scope is illustrated in Figure 6.

From this perspective, sexual harassment against disabled individuals constitutes a serious violation of fundamental Islamic values. Maqāṣid al-Sharı̄'ah, which prioritizes the protection of religion, life, intellect, lineage, and property, underscores the dignity and integrity of every human being, including disabled people.

Sexual harassment violates the protection of life and dignity, both of which are regarded as sacred in Islam. Any violation of the human body, including sexual harassment, is considered a grave sin (Musyafa'ah et al., 2023; Setiani et al., 2017; Syafa'at and Gassing, 2024). Such acts inflict physical and psychological harm on victims, contradicting the core principles of Maqāṣid al-Sharı̄'ah, which seeks to safeguard health and wellbeing.

Additionally, Maqāṣid al-Sharı̄'ah emphasizes the protection of intellect and mental wellbeing (Norman and Ruhullah, 2024). Sexual harassment inflicts psychological trauma, disrupting victims' cognitive and emotional stability. Thus, safeguarding mental health aligns with Maqāṣid al-Sharı̄'ah's commitment to preserving intellect and wellbeing. Sexual harassment against any individual is unacceptable as it contradicts the core objectives of Maqāṣid al-Sharı̄'ah, which seek to uphold honor, stability, and social harmony.

**Figure 7:**
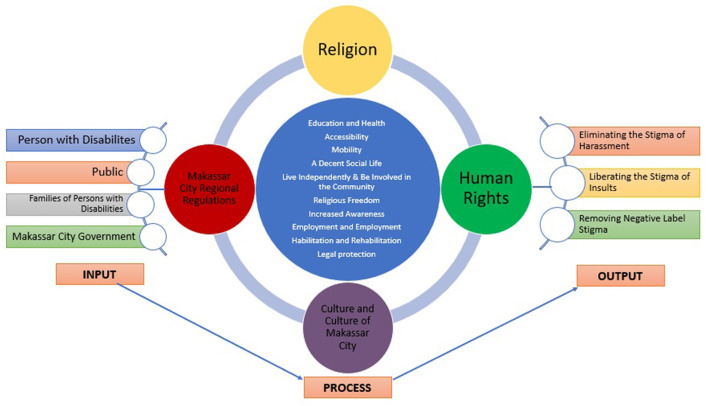
Addressing stigma against disabled people using Maqāṣid al-Sharı̄'ah, Indonesia.

As illustrated in Figure 6, the focus of Maqāṣid al-Sharı̄'ah has evolved from mere protection to community development and empowerment. Similarly, the status of disabled people is shifting from passive protection to active development. The present study applies Jasser Auda's expanded Maqāṣid al-Sharı̄'ah framework to addressing stigma against disabled people, as outlined in Figure 7.

Figure 7 presents a systemic approach to reducing stigma. The inputs in this framework include disabled people, their families, the general public, and the Makassar City Government. The processes involve integrating education, accessibility, and cultural practices, reinforced by local regulations, to foster inclusion and dignity. The outputs aim to eradicate stigma, eliminate harassment, and remove negative labels attached to disabled individuals. These objectives are aligned with the Maqāṣid al-Sharı̄'ah principles of protecting life, intellect, and dignity.

A crucial factor in mitigating stigma is the role of families. While family support can act as a protective barrier against discrimination, societal pressure often compels families to inadvertently contribute to exclusion. This underscores the importance of family-centered interventions, including education, counseling, and empowerment programs. Moreover, religious leaders and communities must actively promote inclusive values based on Maqāṣid al-Sharı̄'ah to reshape societal norms and attitudes toward disabled people.

The implications of this study span several domains. Disability rights laws must integrate Maqāṣid al-Sharı̄'ah principles to ensure alignment with both Islamic and international standards. Increased oversight and accountability are critical to enforcing anti-discrimination measures. Incorporating disability rights education based on Maqāṣid al-Sharı̄'ah into curricula and public campaigns is vital to challenging stereotypes and fostering inclusivity. Religious leaders should interpret and disseminate Islamic teachings on justice and compassion to promote inclusion. Support networks within mosques and Islamic organizations can provide resources for disabled individuals and their families. Counseling and training for families can create supportive home environments. Community-based initiatives must encourage interaction and reduce isolation, fostering mutual understanding between individuals with and without disabilities. Expanding studies to other Muslim-majority regions in Indonesia and assessing the long-term impact of interventions can provide valuable insights for national policies. This integrative approach, grounded in the Maqāṣid al-Sharı̄'ah framework, provides a comprehensive pathway for addressing stigma, ensuring justice, and promoting dignity for disabled individuals in Makassar.

## Conclusion

This research highlights the persistent stigma against disabled people in Makassar, particularly in the form of harassment, insults, and negative labeling. Despite existing legal frameworks, these stigmas continue to marginalize disabled people, undermining their rights and dignity. The study underscores the need for comprehensive strategies that align with the principles of Maqāṣid al-Sharı̄'ah, which emphasize the protection of life, dignity, and intellect.

A key recommendation is the implementation of public education and awareness programs that focus on the rights of disabled people, with the aim of fostering a more inclusive and supportive environment. Additionally, stronger legal enforcement is needed to ensure that disabled people have equal access to education, employment, and other essential services. These measures are crucial for reducing stigma and promoting social justice.

Furthermore, the study calls for increased involvement from both governmental in supporting disabled people. This includes providing resources for skill development, advocating for policy changes, and creating platforms for disabled people to voice their concerns. One of the most important recommendations is to ensure that disabled people are given a voice not only in research but also in all aspects of social life. By actively involving them in decision-making processes, society can move toward a more inclusive and equitable future, where the rights and dignity of all individuals are respected and upheld.

## Data Availability

The original contributions presented in the study are included in the article/supplementary material, further inquiries can be directed to the corresponding author.
